# Validity of a self-reported diagnosis of depression among participants in a cohort study using the Structured Clinical Interview for DSM-IV (SCID-I)

**DOI:** 10.1186/1471-244X-8-43

**Published:** 2008-06-17

**Authors:** Almudena Sanchez-Villegas, Javier Schlatter, Felipe Ortuno, Francisca Lahortiga, Jorge Pla, Silvia Benito, Miguel A Martinez-Gonzalez

**Affiliations:** 1 Department of Clinical Sciencies, University of Las Palmas de Gran Canaria, Spain; 2 Department of Preventive Medicine and Public Health, University of Navarra, Spain; 3 Department de Psychiatry and Medical Psychology, University of Navarra, Spain

## Abstract

**Background:**

Depression assessment in population studies is usually based on depressive symptoms scales. However, the use of scales could lead to the choice of an arbitrary cut-off point depending on the sample characteristics and on the patient diagnosis. Thus, the use of a medical diagnosis of depression could be a more appropriate approach.

**Objective:**

To validate a self-reported physician diagnosis of depression using the Structured Clinical Interview for DSM-IV (SCID-I) as Gold Standard and to assess the factors associated to a valid self-reported diagnosis.

**Methods:**

The SUN Project is a cohort study based on university graduates followed-up through postal questionnaires. The response to the question included in the questionnaire: Have you ever been diagnosed of depression by a physician? was compared to that obtained through the SCID-I applied by a psychiatrist or a clinical psychologist. The percentages of confirmed depression and non-depression were assessed for the overall sample and according to several characteristics. Logistic regression models were fitted to ascertain the association between different factors and a correct classification regarding depression status.

**Results:**

The percentage of confirmed depression was 74.2%; 95% confidence interval (95% CI) = 63.3–85.1. Out of 42 participants who did not report a depression diagnosis in the questionnaire, 34 were free of the disease (%confirmed non-depression = 81.1%; 95% CI = 69.1–92.9). The probability of being a true positive was higher among ex-smokers and non-smokers and among those overweight or obese but the differences were not statistically significant.

**Conclusion:**

The validity of a self-reported diagnosis of depression in the SUN cohort is adequate. Thus, this question about depression diagnosis could be used in further investigations regarding this disease in this graduate cohort study.

## Background

Depression is a serious public health concern in developed countries. Unipolar depressive disorders effects on DALYs (Disability Adjusted Life Years) worlwide [1] have been recently highlighted by the World Health Organization: it accounts for 8% of total DALYs in the Americas and for 6.1% in Europe [2].

However, in spite of the important advance of the psychopharmacology, a proportion of depressed patients are refractory to therapy, adverse effects of medication are frequent and there are difficulties to comply with prescribed treatments. For that reason, a preventive approach is vital, identifying those factors which could decrease depression incidence in large population studies. For example, diet, anthropometry and life-style factors such as smoking or physical activity have been associated to depression in several epidemiological studies [3-7]. However, the large sample size analyzed in these population studies such as prospective cohort studies makes generally necessary the use of questionnaires to collect information. So, exposure and outcome assessment could be partially biased.

Depression assessment in population studies is usually based on depressive symptoms scales. However, the choice of a cut-off point is arbitrary in these scales. This cut-off point usually depends on the sample characteristics (age, pathology, educational level) and on the patient diagnosis (type of depression, seriousness of the problem). Thus, the use of a self-reported medical diagnosis of depression could be a more appropriate approach to reduce misclassification problems in epidemiological studies. In fact, some longitudinal studies have used a self-reported diagnosis to assess different outcomes including depression [4,8].

The aim of our study was to assess the validity of a self-reported physician diagnosis of depression collected through the use of a questionnaire in a sub-sample of the participants of the Seguimiento Universidad de Navarra (SUN) Project using the Structured Clinical Interview for DSM-IV (SCID-I) as the gold standard.

## Methods

### Sample

The SUN Study is a dynamic cohort study based on university graduates. A detailed description of its methods has been published elsewhere [9]. All graduates of University of Navarra and members of professional associations from several Spanish regions have received an invitation letter to the study. Information is collected using self-administered questionnaires sent by postal mail every two years. The questionnaires include questions regarding medical diagnoses such as cardiovascular disease, obesity or depression. The recruitment of participants started in December 1999 and it is permanently on-going as this is a dynamic cohort study. The study was approved by the Human Research Ethical Committee at the University of Navarra. Voluntary completion of the first self-administered questionnaire was considered to imply informed consent.

### Questionnaires

The baseline questionnaire gathered information about sociodemographic and clinical variables and lifestyle factors, and included a previously validated food frequency questionnaire [10]. Participants were asked whether they had ever received a depression diagnosis by a physician. The first (2-years of follow-up) and the second questionnaires (4-years of follow-up) enquired about new depression diagnosis by a physician since the last questionnaire.

### Gold Standard

The SCID-I is a clinician-administered, semi structured interview for use with psychiatric patients or with non patient community subjects who are undergoing evaluation for psychopathology [11]. The SCID-I is the most user-friendly of the clinician-administered interviews and makes diagnoses according to the current DSM. Its main body consists of nine diagnostic modules, Mood Episodes and Mood Disorders Differential included. Interviewer may choose to eliminate one or more modules to selectively focus only on areas of the greatest diagnostic interest. The interview provides required probe questions and suggested follow-up questions. Liberal use or "skip-out" directions are employed when a subject fails to meet a critical criterion required for a particular disorder. The SCID-I had a reusable administration booklet with required questions, suggested follow-up questions, and diagnostic criteria. Diagnoses are made by the interviewer during the course of the interview; no separate scoring algorithm or program is required. The ideal SCID-I interviewer is someone with enough clinical experience and knowledge of psychopathology and psychiatric diagnosis to conduct a diagnostic interview without an interview guide. The reliability and validity of the SCID-I for DSM-III-R has been reported in several studies; although the number of available studies for the DSM-IV is lower.

### Validation study

We selected 256 cases of self-reported depression. This number corresponds to the totality of the self-reported cases of depression residing in Navarra and surrounding regions (País Vasco, Aragón and La Rioja) which had been collected at baseline and during the first and second follow-up questionnaires up to January 2006. We decided to select cases form Navarra's surrounding regions because the number of cases of depression reported in Navarra was not enough to reach our objectives. Moreover, we do not expect that sample characteristics differ substantially among the selected regions.

In addition, we selected a random sample of 181 participants from Navarra without a self-reported physician diagnosis of depression in any questionnaire (at baseline or in the follow-up questionnaires) at that date and who had not participated in other validation studies.

We sent a letter inviting participants to carry out a psychological exam by a psychiatrist or a clinical psychologist at the University Clinic to assess possible medical diagnoses reported by them in different study questionnaires. The letter was accompanied by a list of questions about his/her response regarding medical diagnoses (including depression) in the SUN questionnaires. A contact information form (participant telephone, and e-mail address) and a postage-paid envelope were also included in the mailing.

One hundred and thirty three subjects accepted to participate in the validation study, 85 subjects with a self-reported physician diagnosis of depression (33.2%) and 48 without a self-reported diagnosis of the disease (26.5%).

After the participant reported his/her interest in participating, an appointment with the psychiatrist or the clinical psychologist was made. A small number of the participants were agreed to participate but they reported a lack of time to attend the University Clinic. For that reason, some of them were interviewed by telephone (n = 8), others sent their medical reports regarding depression by postal mail (n = 2) or those who are normally attended at the University Clinic permitted the diagnosis confirmation checking their medical reports by our specialists (n = 5).

At the time of the interview the psychiatrist was unaware of the depression status of the participants according to the questionnaire. Moreover, the participants knew that they would have a psychiatric exam but they were unaware of the exam intention.

Twenty nine subjects (23 with depression and 6 without depression) were not contacted although they agreed to take part in the study. The main reason was that they were abroad. Thus, finally, a total of 104 participants participated in the validation study. Participation among depressed and non-depressed participants was 24.2% and 23.3% respectively.

### Definition of depression

If a participant had reported a physician diagnosis of depression either in the baseline questionnaire or in one of the two follow-up questionnaires, he/she was classified as a participant with a self-reported depression. Otherwise, he/she was considered as non-depressed.

A participant was considered as a true depressed subject if he/she was diagnosed as depressed by one of the psychiatrists or clinical psychologists of the study though the SCID-I.

We defined a participant as a true positive when he/she reported a physician diagnosis of depression in one of the questionnaires (at baseline or in the follow-up) and one of our psychiatrists agreed the diagnosis though the SCID-I.

### Statistical analysis

Pearson's chi squared test or Mann-Whitney U test were used to describe the distribution of several anthropometric, sociodemographic and life-style related characteristics according to self-reported depression status.

The proportion of confirmed cases of depression was calculated as the number of those who reported a physician diagnosis of depression and had depression according SCID-I, divided by all those who reported a physician diagnosis of depression. In the same way, the proportion of non depressed was calculated as the number of subject who did not report a physician diagnosis of depression and were not depressed according the gold standard divided by the total of subjects who did not report a physician diagnosis of depression. Differences in the proportion of confirmed depression and confirmed non-depression according different variables such as age or smoking status were evaluated though Fisher exact tests.

To calculate sensitivity and specificity of the self-reported physician diagnosis of depression the expected distribution of true and false positives and negatives in the sampled population was estimated though the sampling fractions and the observed percentages of confirmed diagnoses. Thus, true prevalence of depression in that population was ascertained. Confidence intervals for that prevalence were estimated using the suggested approach by Cochran for stratified sampling [12].

Logistic regression models were fit to ascertain the association between being correctly classified by the questionnaire (either a true positive or a true negative) and several variables (age, gender, smoking habit, physical activity and body mass index).

## Results

Sixty two subjects with a self-reported physician diagnosis of depression and 42 subjects without the diagnosis were included in the validation study.

Table 1 shows the main characteristics of the participants according self-reported depression status. The mean age was 43.5 (S.D.:12.4) for those with a positive report and 42.7 (S.D.:10.4) for those with a negative one. The proportion of women was higher among subjects with a self-reported diagnosis of depression than among non depressed participants. On the other hand, subjects without a self-reported diagnosis of the disease were physically more active. Nevertheless, there were not statistically significant differences among subjects with and without self-reported diagnosis of depression regarding the sociodemographic, anthropometric and life-style related characteristics.

**Table 1:** Characteristics [mean (Standard deviation)] of the participant in the validation study according self-reported diagnosis of depression.

	**Self-reported diagnosis (n = 62)**	**No Self-reported diagnosis (n = 42)**	**p**
**Age (years)**	43.5 (12.4)	42.7 (10.4)	0.96*
**Gender**			
% Women	77.4	61.9	0.09^#^
**Smoking status (%)**			
Current smoker	27.9	16.7	
Non smoker	39.3	50.0	0.37^#^
Ex – smoker	32.8	33.3	
**Body Mass Index (Kg/m^2^)**	23.3 (4.4)	23.7 (3.5)	0.29*
**Physical activity during leisure time**	25.3 (25.7)	32.8 (28.6)	
(METs/h-week)			
**N° questionnaire (%)δ**			
Baseline	66.1		
2-years follow-up	25.8		
4-years follow-up	8.1		

* Mann-Whitney U test.

^# ^Pearson's chi squared test.

δ Self-reported diagnosis of depression can be collected though baseline questionnaire first follow-up questionnaire (2-years follow-up) or second follow-up questionnaire (4-years follow-up).

There were 46 true positives of the 62 self-reported cases of depression (major depressive episode = 42%; adaptative disorders = 30%; dysthymia = 14%, others = 14%). Thus, the percentage of confirmed diagnosis of depression was 74.2% (95% CI = 63.3–85.1). There were 34 true negatives of the 42 subjects who did not report a depression diagnosis (% confirmed non depression subjects = 81%; 95% CI = 69.1–92.9). Table 2 shows the distribution of the percentage of confirmed depression and non-depression according the main characteristics of the sample. The proportion of confirmed depression was higher among men, younger people and subjects with overweight or obesity. The same pattern was found for the proportion of confirmed non-depression although, for both, the differences were not statistically significant.

**Table 2:** Depression status and validity of self-reported physician diagnosis of depression according different variables

	**N (%)**	**% Confirmed depression**	*p	**% Confirmed non depression**	*p
Total	104 (100)	74.2 (63.3–85.1)		81.0 (69.1–92.9)	
Age					
<45	63 (60.6)	81.6 (69.3–93.9)	0.137	84.0 (69.6–98.4)	0.694
≥45	41 (39.4)	62.5 (43.1–81.9)		76.5 (56.3–96.7)	
Gender					
Men	30 (28.8)	78.6 (57.1–100)	1.000	87.5 (71.3–100)	0.688
Women	74 (71.2)	72.9 (60.3–85.5)		76.9 (60.7–93.1)	
Current smoking					
Yes	24 (23.3)	58.8 (35.4–82.2)	0.116	42.9 (6.2–79.6)	0.017
No	79 (76.7)	79.5 (67.6–91.4)		88.6 (78.1–99.1)	
Physical activity during leisure time (METs-h/week)					
<20	55 (53.9)	85.3 (73.4–97.2))	0.021	76.2 (58.0–94.4)	0.697
≥20	47 (46.1)	57.7 (38.7–76.7)		85.7 (70.7–100)	
Body Mass Index (Kg/m^2^)					
<25	69 (66.3)	69.0 (55.0–83.0)	0.226	77.8 (62.1–93.5)	0.689
≥25	35 (33.7)	85.0 (69.4–100)		86.7 (69.5–100)	

*Fisher exact test

The lifetime prevalence of depression in the population up to the completion of the baseline questionnaire was 26.1%. The expected distribution of true and false positives and negatives was as following: we expected 694 true positives from 935 participants who reported a physician diagnosis of depression at baseline or in the follow-up questionnaires. Similarly, the number of true negatives in the source population would be 5126 from 6329 participants who reported not to have had a physician diagnosis of depression. Thus, the estimated sensitivity and specificity for our population were 0.37 and 0.96 respectively.

Subjects more physically active had a decreased probability to be correctly classified as depressed (OR = 0.24; 95% CI = 0.07–0.80) but, statistically significant differences were apparent only in the crude analysis (Table 3).

**Table 3:** Relationship between to be a true positive and sociodemographic, anthropometric and life-style related variables (subject who did not report a physician diagnosis of depression were excluded = 42 subjects).

	**Crude OR**	**IC 95%**	**Adjusted OR^#^**	**IC 95%**
Age				
<45	1 (ref.)		1 (ref.)	
≥45	0.38	0.12–1.21	0.30	0.07–1.26
Gender				
Men	1 (ref.)		1 (ref.)	
Women	0.73	0.18–3.06	0.87	0.08–10.03
Current smoking				
Yes	1 (ref.)		1 (ref.)	
No	2.72	0,81–9.15	3.41	0.81–14.32
Physical activity during leisure time (METs-h/week)				
<20	1 (ref.)		1 (ref.)	
≥20	0.24	0.07–0.80	0.27	0.07–1.09
Body Mass Index (Kg/m^2^)				
<25	1 (ref.)		1 (ref.)	
≥25	2.54	0.63–10.21	4.03	0.41–39.61

^# ^Adjusted for the variables in the table.

Non-smokers had a statistically significant higher probability to be correctly classified as free from depression (OR = 10.33; 95% CI = 1.67–64.0) (Table 4).

**Table 4:** Relationship between to be a true negative and sociodemographic, anthropometric and life-style related variables (subject who reported a physician diagnosis of depression were excluded = 62 subjects).

	**Crude OR**	**IC 95%**	**Adjusted OR^#^**	**IC 95%**
Age				
<45	1 (ref.)		1 (ref.)	
≥45	0.62	0.13–2.91	0.68	0.10–4.77
Gender				
Men	1 (ref.)		1 (ref.)	
Women	0.48	0.08–2.71	0.72	0.10–5.23
Current smoking				
Yes	1 (ref.)		1 (ref.)	
No	10.33	1.67–64.00	10.37	1.27–84.41
Physical activity during leisure time (METs-h/week)				
<20	1 (ref.)		1 (ref.)	
≥20	1.88	0.39–9.12	1.23	0.13–11.21
Body Mass Index (Kg/m^2^)				
<25	1 (ref.)		1 (ref.)	
≥25	1.86	0.33–10.62	2.90	0.29–29.36

^# ^Adjusted for the variables in the table.

## Discussion

Among the subjects who self-reported a physician diagnosis of depression in at least one of the questionnaires used in this cohort study, the percentage of confirmed diagnosis of depression was high. The percentage of subjects without real depression who had responded negatively to the questions regarding depression status in the questionnaires was even higher.

However, when several sample characteristics of the sample were analyzed, differences in the degree of confirmation regarding depression status were found. A more favourable response (better agreement between self-reported diagnosis and the gold standard) regarding the presence of depression was found for men, younger subjects, non-smokers, overweight and obese subjects and participants with a low level of physical activity during leisure time; but only physical activity was statistically associated with a correct positive classification of depression. Due to the high number of characteristics analysed, we can not exclude the possibility that some spurious associations could have occurred. However, higher prevalence of depression should lead to a higher proportion of confirmed depression. Depression has been reported to be inversely associated with physical activity in several epidemiological studies [5,6]. Thus, a higher prevalence of depression or the presence of depressive symptoms (asthenia, tiredness) is expected among sedentary subjects.

In the same way, the proportion of correctly identifying a subject as free from depression was higher among non smokers. Smoking has also been associated to depression [7].

The validity of the self-reported diagnosis of different diseases has been sufficiently studied in several populations in which the educational level of the participants was as high as it is in the SUN Project. The SUN Project uses similar methodologies to those of large cohort studies such as Nurses' Health Study or the Health Professionals Follow-up Study. In these cohort studies disease status is collected though questionnaires sent by mail every 2 years as we do. Predictive values obtained in their validation studies are generally high [13,14]. The SUN Study has also assessed the validity of some questions included in the questionnaires regarding several diseases or conditions like hypertension [15] or body mass index [16] obtaining good results.

Most of the population studies which have analyzed depression have used depressive symptom scales to ascertain the disease: Diagnostic Inventory for Depression [17], Center for Epidemiological Studies Depression Scale [18], Hospital Anxiety and Depression Scale [19] or Beck Depression Inventory [20]. In the majority of the cases, the Structured Clinical Interview for the Diagnostic and Statistical Manual of Mental Disorders, fourth edition (SCID-I) was used as gold standard to assess validity of the scales. However, an important limitation of the use of depressive scales is the arbitrary election of a cut-off point to define depression status. The confirmation of the disease for a specialist though a physician interview (categorical definition) is clearly the best option to assess a depressive disorder although, at this moment, it is controversial the use of a categorical classification to define a depressive status. Some authors defend the idea of a dimensional classification in which depression could be defined as a continuum ranged from absence of any symptom to the presence of symptoms with maximum intensity. On the other hand, under-diagnosis of depression occurs in the 44.3% of the patients coming to a primary care center [21]. A recent study found a sensitivity of 40% and a specificity of 87% for a general practitioner diagnosis of a major depressive disorder as compared with that found through the SCID-I made by a psychiatrist [22]. Therefore, it was necessary to carry out a validation study to rule out disease under-estimation in the questionnaires among our participants due to under-diagnosis. This situation should lead to a decrease of the proportion of confirmed non-depression.

The difficulty to classify depression correctly could have decreased the proportion of confirmed depression in the validation study. There are problems in differential diagnosis because depressive experiences vary from individual to individual. The co-occurrence of symptoms of anxiety and depression is very common. In a recent community study, the 56.3% of the sample with current major depressive disorder had also another mental disorder [23]. On the other hand, some of the participants did not have a specific clinical report, only a verbal diagnosis from their physician. Thus, the depressive symptoms secondary to other primary psychiatric disorders or other pathology could be easily named depression.

We acknowledge that participation in the validation study was low (24.2% and 23.3% for depressed and non-depressed subjects, respectively) and therefore a selection bias cannot be excluded, conferring an artificially high validity to our results. To prevent this bias, we put special care to blind participants about the aim of the psychiatrist interview. Thus we tried to avoid that those participants who may be aware of incorrectly classifying themselves as depressed may be embarrassed of participating. Moreover, when we compared the socio-demographic characteristics of participants (n = 104) and non-participants (n = 333) we did not find any systematic difference regarding key variables (sex, age, smoking status, BMI, or physical activity) in their baseline assessment.

Finally, a small proportion of participants were not interviewed face to face by the specialists. They were interviewed by telephone, they sent their medical report by postal mail or they permitted the access to their reports at the University Clinic. Although a participant could lie or modify his/her responses when the interviewer is not present, the validity of depression assessment though the use of telephone has been demonstrated [24]. Whereas the assessment of the disease could have been modified because of the use of a telephone interview, we do not expect any change regarding depression assessment using existing medical reports.

## Conclusion

In conclusion, self-reported diagnosis of depression in the SUN study is adequate and can be used in this large cohort to assess depression status. The proportion of confirmed depression was higher among sedentary subjects and the proportion of confirmed non-depression among non smokers.

## Abbreviations

DSM-IV: Diagnostic and Statistical Manual of Mental Disorders, Fourth Edition; SCID-I: Structured Clinical Interview Diagnostic for DSM-IV Axis-I Disorders; SUN: Seguimiento Universidad de Navarra, University of Navarra Follow-up Study; 95% CI: 95% Confidence Interval; DALYs: Disability Adjusted Life Years; S.D.: Standard Deviation.

## Competing interests

The authors declare that they have no competing interests.

## Authors' contributions

AS-V and MAM-G were responsible for study design, data collection and obtaining funding. AS-V and SB were responsible for the data analysis. JS, FO, FL and JP carried out the psychiatric interviews and confirmed the medical reports of the participants. AS-V was responsible the first drafting of the manuscript. All authors have revised the manuscript and read and approved the final manuscript.

## Pre-publication history

The pre-publication history for this paper can be accessed here:



## References

[B1] World Health Organization (WHO) (2003). World Health Report 2003. Shaping the future.

[B2] Üstün TB, Syuso-Mateos JL, Chatterji S, Mathers C, Murray CJL (2004). Global burden of depressive disorders in the year 2000. British Journal of Psychiatry.

[B3] Sanchez-Villegas A, Henríquez P, Figueiras A, Ortuño F, Lahortiga F, Martínez-González MA (2007). Long chain omega-3 fatty acids intake, fish consumption and mental disorders in the SUN cohort study. European Journal of Nutrition.

[B4] Herva A, Laitinen J, Miettunen J, Veijola J, Karvonen JT, Läksy K, Joukamaa M (2006). Obesity and depression: results from the longitudinal Northern Finland 1966 Birth Cohort Study. International Journal of Obesity (London).

[B5] Sánchez-Villegas A, Ara I, Guillén-Grima F, Bes-Rastrollo M, Varo-Cenarruzabeitia JJ, Martinez-Gonzalez MA (2008). Physical activity, sedentary index and mental disorders in the SUN cohort study. Medicine and Science in Sports and Exercise.

[B6] Harris AH, Cronkite R, Moos R (2006). Physical activity, exercise coping, and depression in a 10-year cohort study of depressed patients. Journal of Affective Disorders.

[B7] Sanchez-Villegas A, Serrano-Martínez M, Alonso A, De Irala J, Tortosa A, Martínez-González MA (2008). Role of the tobacco use on the depression incidence in the SUN cohort study after six years of follow-up. Medicina Clinica (Barc).

[B8] Varraso R, Jiang R, Barr RG, Willett WC, Camargo CA (2007). Prospective study of cured meats consumption and risk of chronic obstructive pulmonary disease in men. American Journal of Epidemiology.

[B9] Martinez-Gonzalez MA, Sanchez-Villegas A, De IJ, Marti A, Martinez JA (2002). Mediterranean diet and stroke: objectives and design of the SUN project. Seguimiento Universidad de Navarra. Nutritional Neuroscience.

[B10] Martin-Moreno JM, Boyle P, Gorgojo L, Maisonneuve P, Fernandez-Rodriguez JC, Salvini S, Willett WC (1993). Development and validation of a food frequency questionnaire in Spain. International Journal of Epidemiology.

[B11] First MB, Spitzer RL, Gibbon M, Williams JBW (1999). Entrevista Clínica Estructurada para los Trastornos del Eje I del DSM-IV (SCID-I) SCID-I (versión clínica) Masson S A Barcelona (Spain).

[B12] Cochran WG, Cochran WG (1977). Stratified random sample. Sampling Techniques.

[B13] Fung TT, Hu FB, Pereira MA, Liu S, Stampfer MJ, Colditz GA, Willett WC (2002). Whole-grain intake and the risk of type 2 diabetes: a prospective study in men. American Journal of Clinical Nutrition.

[B14] Manson JE, Colditz GA, Stampfer MJ, Willett WC, Krolewski AS, Rosner B, Arky RA, Speizer FE, Hennekens CH (1991). A prospective study of maturity-onset diabetes mellitas and risk of coronary heart disease and stroke in women. Archives of Internal Medicine.

[B15] Alonso A, Beunza JJ, Delgado-Rodríguez M, Martínez-González MA (2005). Validation of self reported diagnosis of hypertension in a cohort of university graduates in Spain. BMC Public Health.

[B16] Bes-Rastrollo M, Pérez Valdivieso JR, Sánchez-Villegas A, Alonso A, Martínez-González MA (2005). Validación del peso e índice de masa corporal auto-declarados de los participantes de una cohorte de graduados universitarios. Revista Española de Obesidad.

[B17] Zimmerman M, Sheeran T, Young D (2004). The Diagnostic Inventory for Depression: a self-report scale to diagnose DSM-IV major depressive disorder. Journal of Clinical Psycology.

[B18] Soler J, Pérez-Sola V, Puigdemont D, Pérez-Blanco J, Figueres M, Álvarez E (1997). Estudio de validación del Center for Epidemiologic Studies-Depression (CES-D) en una población española de pacientes con trastornos afectivos. Actas luso-españolas de neurología, psiquiatría y ciencias afines.

[B19] Herrero MJ, Blanch J, Peri JM, De Pablo J, Pintor L, Bulbena A (2003). A validation study of the hospital anxiety and depression scale (HADS) in a Spanish population. General Hospital Psychiatry.

[B20] Bonilla J, Bernal G, Santos A, Santos D (2004). A revised Spanish version of the Beck Depression Inventory: psychometric properties with a Puerto Rican sample of college students. Journal of Clinical Psycology.

[B21] Gabarron Hortal E, Vidal Royo JM, Haro Abad JM, Boix Soriano I, Jover B, Arenas Prat M (2002). Prevalencia y detección de desórdenes depresivos en atención primaria. Atencion Primaria.

[B22] Lowe B, Spitzer RL, Grafe K, Kroenke K, Quenter A, Zipfel S, Buchholz C, Witte Herzog W (2004). Comparative validity of three screening questionnaires for DSM-IV depressive disorders and physicians' diagnoses. Journal of Affective Disorders.

[B23] Blazer DG, Kessler RC, McGonagle KA (1994). The prevalence and distribution of major depression in a national community sample: the national comorbidity survey. American Journal of Psychiatry.

[B24] Carrete P, Augustovski F, Gimpel N, Fernandez S, Di Paolo R, Schaffer I, Rubinstein F (2001). Validation of a telephone-administered geriatric depression scale in a Hispanic elderly population. Journal of General Internal Medicine.

